# The Influence of Professional Identity, Job Satisfaction, and Work Engagement on Turnover Intention among Township Health Inspectors in China

**DOI:** 10.3390/ijerph15050988

**Published:** 2018-05-14

**Authors:** Wenjie Zhang, Hongdao Meng, Shujuan Yang, Danping Liu

**Affiliations:** 1 Department of Health and Social Behavior, School of Public Health, Sichuan University, Chengdu 610041, China; howwenj@163.com (W.Z.); rekiny@126.com (S.Y.); 2 School of Aging Studies, College of Behavioral & Community Sciences, University of South Florida, Tampa, FL 33620, USA; meng@usf.edu

**Keywords:** public health workforce, health inspectors, turnover intention, job satisfaction, professional identity, work engagement, China

## Abstract

Health inspectors are part of the public health workforce in China, and its shortage has been identified as an urgent priority that should be addressed. Turnover is one of the main contributors to the shortage problem. This research assessed the influence of professional identity, job satisfaction and work engagement on turnover intention of township health inspectors and explored the intermediary effect of job satisfaction and work engagement between professional identity and turnover intention among township health inspectors in China. Data were collected from 2426 township health inspectors in Sichuan Province, China. We used structural equation modeling (SEM) to test the hypothesized relationship among the variables. Results showed that a total of 11.3% of participants had a high turnover intention and 34.0% of participants had a medium turnover intention. Job satisfaction had a direct negative effect on turnover intention (β = −0.38, *p* < 0.001), work engagement had a direct negative effect on turnover intention (β = −0.13, *p* < 0.001), and professional identity had an indirect negative effect on turnover intention through the mediating effect of job satisfaction and work engagement. Our results strongly confirmed that professional identity, job satisfaction and work engagement were strong predicators of turnover intention. According to the results, desirable work environment, quality facilities, fair compensation and adequate advancement opportunities should be emphasized to improve job satisfaction. The turnover intention of health inspectors could be reduced through improving professional identity, enhancing job satisfaction and work engagement.

## 1. Introduction

In China, health inspectors are part of the public health workforce who perform comprehensive supervision and inspection works involving the hygienic status of public places, drinking water safety, school health, licensure of health care providers, occupational health and safety, family planning and other functions [1]. Based on this, health inspectors conduct a much broader scope of work as compared to other countries and play a critical role in maintaining public health order and protecting people’s health rights in China [2]. The Health and Family Planning Supervision and Law Enforcement Agency (HFPSaLIA, China) was mandated to recruit a sufficient number of skilled workers. However, worker shortages have been identified as an urgent priorities that should be addressed. The former Ministry of Health in China required 1–1.5 health inspectors per 10,000 permanent residents in the area [3]. Studies drawing data from more than 20 provinces have found the health inspectors were less than one per 10,000 in eastern, central and western areas of China [4,5]. 

Due to the broad scope of their job responsibilities, health inspectors are required to be trained in diverse fields including medicine, law, and management. With a large population and territory to manage and heavy workload, turnover has been cited as one of the main contributors to the shortage problem. At the same time, HFPSLIA are under increasing threat from the turnover of health inspectors, especially those with high-quality talents [6]. The average turnover of health inspectors was increased from 0.84% in 2006 to 1.42% in 2009 in China [7]. In addition, many health inspectors had an intention to leave their current job [6]. As a result, the turnover behavior of health inspectors not only leads to workforce shortage but also causes a decline in human resource quality.

Turnover intention refers to the probability that an employee voluntarily leaves his or her job in the period ahead [8]. Past research has demonstrated that turnover intention is the principal cognitive precursor of turnover behavior with great explanatory power [9,10]. After March and Simon proposed the earliest participant determination model on turnover in 1958, scholars constructed different theoretical models around the turnover [8,10,11,12]. A large number of studies have analyzed the influencing factors of turnover intention, mainly including external environment factors such as local level of employment and alternative job opportunities, internal individual factors like work ability, and the most widely studied job-related factors such as organizational justice, work stress, affective commitment, work hours and so on, different combinations of these factors were always incorporated into the studies to explore the combined effect on turnover intention [13,14,15,16,17].

Job satisfaction, an individual’s affective reaction to a job based on a comparison of practical with ideal outcomes, has been frequently considered as a predicator of turnover in health care providers [18,19]. A large number of studies have found a negative correlation between job satisfaction and turnover intention, a study came to the same conclusion that the low satisfaction of doctors in Australia was associated with the intention to leave the medical workforce [20]. Workers with high job satisfaction also had lower turnover intention in samples of Slovenia and Austria [21]. Components of job satisfaction associated with turnover intention involving many aspects and those frequently assessed include income, supervisors, colleagues, organizational factors and work environment [22]. In addition, job satisfaction is also a moderator of the relationship between other factors and turnover intention, for example, the subjective social status exerted a significant indirect effect on turnover intention through job satisfaction among Chinese nurses [23].

Work engagement is defined as a stable and positive emotional activation state of employees including three dimensions of vigor, dedication and absorption [24]. Individual characteristics such as emotional intelligence and achievement striving are considered to be antecedent variables that affect work involvement [25,26]. Organizational support, work resources and other work characteristics also affect work engagement [27,28]. Accordingly, the outcome variables of work engagement are also divided into individual and organization parts, turnover intention is one of the individual outcomes of work engagement, individuals with high work engagement are less likely to have turnover intention [24,29]. Some studies explored the mediating effect of work engagement. Shahpouri et al. found that job resource and personal resource affected turnover intention through work engagement among female nurses [30]. A study made in South Africa revealed the mediating effect of work engagement between workplace bullying and turnover intention [31]. 

As a self-concept, professional identity refers to the individual’s understanding of the social impact of profession and the significance of individual’s work, it is the psychological basis for people to do their job well and achieve the organizational goal [32]. The development of professional identity is a dynamic process that links the job role to clear self-perceptions including professional interests, skills, aims and values, and it gives meaning and orientation to one’s profession [33]. Professional identity has been considered to be the influencing factor of turnover intention, job satisfaction and work engagement, high professional identity reduced risk of high turnover intention [34]. A study made in Turkey demonstrated the positive effect of professional identity on job satisfaction among nurses [35]. Chris Bothma et al. revealed that professional identity played a positive leading role in work engagement [36]. Most of existing studies of professional identity focus on teachers and health workers [34,37]. 

**Figure 1:**
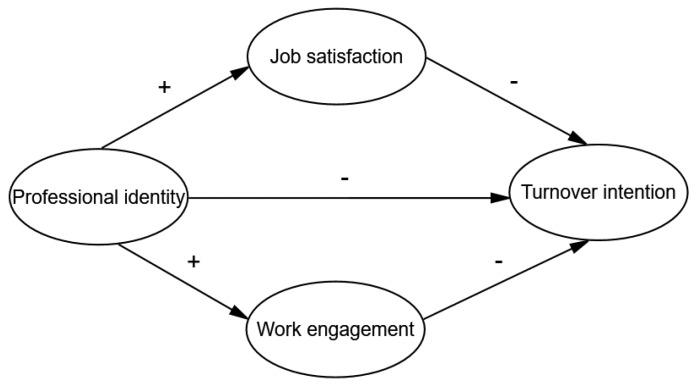
The theoretical model and hypotheses.

Based on the above theoretical analysis and empirical support, we tried to link the relationships among professional identity, job satisfaction, work engagement and turnover intention and hypothesized a double mediator model shown in Table 1 and Figure 1. We assumed that job satisfaction and work engagement directly affect turnover intention, and professional identity affects turnover intention in two ways: directly and indirectly (through job satisfaction and work engagement); this study will further explore the intermediary effect of job satisfaction and work engagement between professional identity and turnover intention. This study is the first to examine the influence of professional identity, job satisfaction and work engagement on turnover intention of township health inspectors in China.

## 2. Materials and Methods 

### 2.1. Setting and Participants

This cross-sectional study was conducted among the township Health Law Enforcement and Supervision Agencies in Sichuan Province, China from August to October in 2017. A multistage stratified random sampling survey was used to acquire the sample. In the first stage, we randomly chose 115 districts and counties from 183 in the entire province. In the second stage, half of the towns were randomly selected from each district and county. In the third stage, the selected towns were asked to randomly choose 2 health inspectors. The data were collected by anonymous questionnaires, consisting of five parts along with a covering letter outlining the survey objective and return methods. The questionnaires were delivered to 115 districts and counties through an official letter by The Health and Family Planning Commission of Sichuan Province, and a total of 2426 randomly-chosen health inspectors filled and returned the questionnaire in the end with an effective response rate of 97%.

### 2.2. Measures

The questionnaire was designed for National Health and Services Survey by an expert panel from National Health and Family Planning Commission of the People’s Republic of China [38]. The questionnaire included five parts, socio-demographic characteristics (gender, age, marital status, major, educational background, years of work), questions related to professional identity, job satisfaction, work engagement and turnover intention.

### 2.3. Professional Identity

The Chinese version of professional identity questionnaire comprised three items: (1) My work has a significant impact on the lives of others, (2) The quality of my work will affect many people, (3) my work is very meaningful and important. Five-point Likert scale ranging from 1 (highly disagree) to 5 (highly agree) was utilized to evaluate all these items, a higher score indicated higher identification of job meaning. The Cronbach’s Alpha coefficients of this questionnaire was 0.831 [38].

### 2.4. Job Satisfaction

Job satisfaction was measured by the Chinese version of the Job Descriptive Index [39] and the Cronbach’s Alpha coefficients was 0.659 [38]. It comprised eight items: the work itself, advancement, compensation, environment, facility, colleagues, superiors and current job. Six-point Likert scale ranging from 1 (highly disagree) to 6 (highly agree) was utilized to evaluate all these items, a higher score indicated higher job satisfaction. 

### 2.5. Work Engagement

Work engagement was measured by the Chinese version of the Utrecht Work Engagement Scale [40] and the Cronbach’s Alpha coefficients was 0.782 [38]. It comprised seventeen items, consisted of 3 scales, work vigor (6 items), work dedication (5 items), and work absorption (6 items). A seven-point Likert scale ranging from 0 (never) to 6 (every day) was utilized to evaluate all these items (reverse scored). Responses were combined into summary scales and a higher score indicated higher work engagement. 

### 2.6. Turnover Intention

The Chinese version of turnover intention questionnaire was developed by Cammannet [41] et al. and Mobley [10] et al. and the Cronbach’s Alpha coefficients was 0.659 [38]. It comprised four items: “Thought of leaving the organization you served now”, “Thought of leaving this industry”, ”Looking for a new job recently”, ”Looking for a new job next year”. Six-point Likert scale ranging from 1 (highly disagree) to 6 (highly agree) was utilized to evaluate all these items, a higher score indicated higher turnover intention. The total score was evenly divided into low, middle and high grades.

### 2.7. Statistical Analysis

Although the questionnaire has been used in The National Health and Services Survey in China, it is the first time to use it among health inspectors. We used exploratory factor analysis (EFA) to evaluate the liability and validity of the whole questionnaire.

Descriptive statistics were used to examine sample demographic characteristics of participants, including gender, age, marital status, education background, major and years of work. Then we undertook a descriptive analysis of participants’ professional identity, job satisfaction, work engagement, and turnover intention, means and standard deviations (SD) were used. Pearson correlation coefficient was used to analyze the correlation of major observed variables of latent constructs.

Structural equation model (SEM) was employed to further test hypothesized relationships among the four dimensions, professional identity, job satisfaction, work engagement, and turnover intention. The SEM using bootstrap maximum likelihood estimation. The fit between the current data and hypothesized model was assessed through several indicators, adjust goodness of fit index (AGFI), a goodness of fit index (GFI), normed fit index (NFI), comparative fit index (CFI), incremental (IFI), and Tucker-Lewis index (TLI) of 0.90 or above, a root mean squared error of approximation (RMSEA) less than 0.08, indicated an acceptable model fit.

### 2.8. Reliability and Validity

Based on the results of EFA, the KMO of this questionnaire was 0.876, where the value greater than 0.70 indicates better possibility of factor analysis. The χ^2^ of Bartlett test of sphericity was 29,813.911 (*p* < 0.001). Factor analysis based on principal components for the dimensions extracted common factors and did orthogonal rotation with varimax procedure, finally extracted 4 factors with eigenvalue greater than 1 and been concluded as professional identity, job satisfaction, work engagement and turnover intention, cumulative explanatory variance explained 69.483%. All the loading values of the items to the corresponding dimensions were greater than 0.53, so the construct validity of the questionnaire was good. The total Cronbach’s Alpha coefficients of factors was 0.744, indicated that the questionnaire has good internal consistency reliability.

## 3. Results

### 3.1. Demographic Characteristics of Participants

Individual socio-demographic characteristics of the 2426 workers are shown in Table 2. In the samples, 52.27% were men and the largest proportion of respondents (34.13%) was in the 40–49 age group, followed by the 30–39 age group (32.03%). The average age of the respondents was 39.78 ± 9.19 years. The majority of respondents were married (87.35%). Most of the respondents had obtained a junior college’s degree (51.07%). More than half of them without professional background (65.79%) and more than half of them work less than 5 years (52.56%).

### 3.2. Descriptive Analysis of Study Variable

The total item scores of professional identities, job satisfaction, work engagement and turnover intention were 11.56 ± 2.36, 36.58 ± 7.12, 62.32 ± 22.09, 10.22 ± 5.49 respectively. The item score of each dimension is shown in Table 3. Based on the score, 274 (11.3%) of health inspectors had high turnover intention and 825 (34.0%) had medium turnover intention. Job satisfaction in advancement, compensation, environment and facility was relatively lower than the other four items with the score of 4.12 ± 1.50, 3.97 ± 1.44, 4.49 ± 1.24, 4.24 ± 1.28 respectively.

### 3.3. Correlations of Study Variables

The Pearson’s correlations for the study variables are shown in Table 4. Job satisfaction had a positive correlation with professional identity and a negative correlation with turnover intention, work engagement had a positive correlation with professional identity and a negative correlation with turnover intention, professional identity had a negative correlation with turnover intention.

### 3.4. Test of Study Model

**Figure 2:**
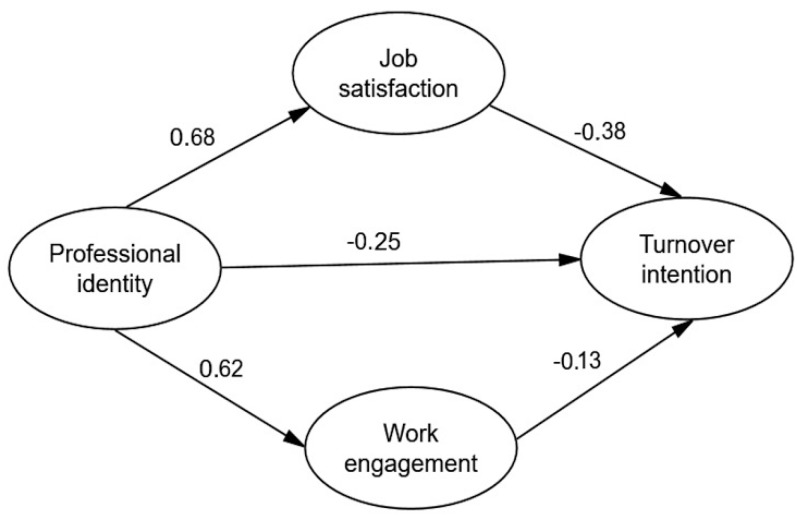
The final model and standardized model paths.

The SEM was established to interlink the four study variables and assess the relationship among them. We fitted the data and the theoretical model through the Generalized least squares and modified the theoretical model according to model fit indices. The final output model was shown in Figure 2 which presented correlation and effect path of the four study variables. The overall model fit indices of the modified hypothetical model were AGFI = 0.918, GFI = 0.940, NFI = 0.956, CFI = 0.960, IFI = 0.960, TLI = 0.952, RMSEA = 0.062, all of them satisfied reference value, suggesting an acceptable model fit.

Bias-corrected bootstrap with 2000 replications using maximum likelihood estimation was employed for each path, the results of the mediation analysis are shown in Table 5. If the 95% CI of the estimation of the mediate effect does not include 0, it means that the mediate effect is statistically significant. Professional identity had a direct positive effect on job satisfaction (β = 0.68, *p* < 0.001). Job satisfaction had a direct negative effect on turnover intention (β = −0.38, *p* < 0.001). Professional identity had a direct positive effect on work engagement (β = 0.62, *p* < 0.001). Work engagement had a direct negative effect on turnover intention (β = −0.13, *p* < 0.001). However, professional identity had no significant direct effect on turnover intention (β = 0.09, *p* = 0.09). The final results supported all hypotheses except Hypothesis 5.

Regarding the path between professional identity and turnover intention, the total effect and indirect effect of this path was statistically significant, which means the mediate effect exists, but the direct effect between this path was not significant, indicating that the model supports the hypothesis that job satisfaction and work engagement have a completely indirect effect between professional identity and turnover intention. Table 6 shows that the 95%CI of the estimation of the two-mediation path does not include 0, indicates that completely indirect effect of job satisfaction and work engagement between professional identity and turnover intention are both statistically significant.

## 4. Discussion

This study is dedicated to exploring the status of turnover intention and the effects of professional identity, work engagement and job satisfaction on turnover intention among township health inspectors in China. The special value of this study is not only the selection of health inspectors as subjects, but also the first time to integrate the four variables in one model.

The results showed that while a small minority (11.3%) had high turnover intention, more than a third (34.0%) of health inspectors had medium turnover intention. The turnover intention rate was higher than that of village doctors in China (36.8%) [42], but similar to those found in a sample of hospital-based physicians in Taiwan (14.5% and 30.0%, respectively) [14]. Overall, 45.3% of health inspectors had medium to high turnover intention and above. The issue of public health workforce shortage is not unique to China as a recent study suggest that more than 40% of US public health workers in state agencies either have plans to retire by 2020 or intend to leave their current organization within 1 year [43]. In addition, our study also investigated the quality of current township health inspectors, the turnover behavior of health inspectors did influence human resource quality. Only 702 (28.94%) of them had university qualifications or above, and there were still 485 (19.99%) of them have less than technical secondary school. More than half (65.79%) of them without major background of medicine, preventive medicine, health administration and law. In addition, because of alarming turnover, more than half (52.56%) of existing health inspectors had less than five years’ work experience. Therefore, more research is urgently needed to explore the key influencing factors related to staff turnover in the township health inspection workforce.

The model proved job satisfaction negatively influenced turnover intention which had been mentioned in other studies. Alsaraireh et al. revealed a statistically-significant negative relationship between job satisfaction and turnover intention among Jordanian nurses in psychiatric units [44]. A discriminant analysis by Lu et al. showed that 38.4% of job satisfaction was correctly classified in predicting intention to leave the organization [45]. In our study, job satisfaction was the most powerful contributors for turnover intention even allowing after both direct and indirect path. The job satisfaction of health inspectors was not bad, but health inspectors had lower satisfaction with advancement, compensation, environment and facilities (Table 3). These factors were consistent with other studies in China; an investigation of health inspectors in Weifang, Shandong province in China found most were dissatisfied with salary and benefits, advancement opportunities and fairness in assignments [46]. 

The model also showed work engagement negatively influenced turnover intention, which was consistent with previous studies. Laschinger found that work engagement was a significant predictor of turnover intention among newly graduated nurses [47]. Workers who engaged in their work put high levels of energy and enthusiasm into what they do, the feeling of self-worth increasing their intention to stay [48]. Based on the motivational process of job demands-resources model, workers translated sufficient job resources into high work engagement and further lead to positive job outcomes both in personal and organizational performance. Even with inadequate job resources, workers may not emerge in high turnover intention because the mediating effect of work engagement [49]. 

The results revealed that professional identity had a positive effect on job satisfaction and work engagement. This conclusion is consistent with other studies [34]. When individuals have a positive identity with their careers, they will devote more energy and enthusiasm to their work, and the dissatisfaction caused by working environment would be eliminated to a certain extent [31]. In addition, if certain aspects of an individual’s identity are consistent with occupation, individuals can maintain a high level of work engagement even in unfavorable working conditions [50]. As a necessary component of legislation construction, health inspection plays an important role in ensuring public health and raising the public health awareness of people, the value of this occupation on improving health status for everyone cannot be ignored [51]. 

All hypotheses of this study were supported except hypothesis 5, that professional identity had no direct effect on turnover intention according to results and it was different from previous studies [34,52]. However, the effect of professional identity on turnover intention was also affirmed, and the most significant finding of this study was that the relationship between professional identity and turnover intention was mediated via job satisfaction and work engagement. The mediating effect revealed that job satisfaction and work engagement were necessary paths for health inspectors to translate the unification of professional and personal value into the possibility of stay. Interventions that can effectively reduce turnover intention of health inspectors should be inspired by this mediating path; one possible way of intervention is to regard high professional identity as the key factor in identifying workers with high job satisfaction and work engagement [53]. Another possible strategy is to promote value consistency between job and self-expectations to enhance the professional identity, which is essential for high work engagement and therefore for low intention to leave [54,55,56,57].

In conclusion, three affecting paths of turnover intention were revealed in our study: professional identity, job satisfaction and work engagement were all strong predicators of turnover intention, especially job satisfaction was the major contributor, due to the fact that professional identity had an indirect effect on turnover intention through job satisfaction and work engagement instead of affecting directly. It suggests that a rather complex mechanism exists in the relationship between professional identity and turnover intention. The direct effect of professional identity on turnover intention should be extensive inquiry among other subjects, and future studies should explore more mediators that may link professional identity to turnover intention. 

The limitations of the study should be addressed. Firstly, despite the SEM being used to determine the relationships among variables, this study still has limitations to draw definite conclusions based on the cross-sectional design. Secondly, we collected the data through the participants’ self-report and returned questionnaires rather than face-to-face investigation.

## 5. Conclusions

This study examined key predictors of intentions to leave one’s job in a large sample of township health inspectors in China. The main contribution of this study was examining the role of professional identity as it relates to turnover intention, work engagement, and job satisfaction. Results confirmed that professional identity exerted a significant indirect effect on turnover intention via job satisfaction and work engagement while job satisfaction and work engagement exerted a significant direct effect. Higher professional identity, job satisfaction and work engagement contribute to reducing turnover intention. These results not only offer a new idea of the reason for township health inspectors’ intentions to leave their jobs but also supply possible new methods for solving the turnover problem. Future research is recommended to construct different models to examine the mechanism of professional identity on turnover intention by introducing other mediator factors.

Based on the results, the policy makers of the supervision system might consider taking a series of measures to improve job satisfaction and work engagement of health inspectors. The dimensions associated with work should be noted, including ensuring a comfortable work environment and ample necessary facilities, providing higher and more rational pay and offering more advancement opportunities. At the same time, measures should be taken to improve health inspectors’ identity that could act effectively on work engagement and job satisfaction. In this way, the township health supervision agency could maintain a stable and dynamic health inspectors group, as needed. 

## Figures and Tables

**Table 1:** The theoretical Hypotheses.

Hypotheses
1. Job satisfaction has a negative effect on turnover intention
2. Work engagement has a negative effect on turnover intention
3. Professional identity has a positive effect on job satisfaction
4. Professional identity has a positive effect on work engagement
5. Professional identity has a direct negative effect on turnover intention
6. Professional identity has an indirect negative effect on turnover intention through the mediating effect of job satisfaction
7. Professional identity has an indirect negative effect on turnover intention through the mediating effect of work engagement

**Table 2:** Demographic characteristics of participants (*n* = 2426).

Socio-Demographic Information	*n*	%
Gender		
Male	1268	52.27
Female	1158	47.73
Age, group		
<30	376	15.50
30∼	777	32.03
40∼	828	34.13
50∼	445	18.34
Marital status		
Unmarried	217	8.94
Married	2119	87.35
Other	90	3.71
Education background		
Lower than senior school	186	7.67
Technical secondary school	299	12.32
Junior college	1239	51.07
University or above	702	28.94
Major		
Clinical medicine	509	20.98
Preventive medicine	23	0.95
Health administration	94	3.87
Law	204	8.41
Other	1596	65.79
Years of work		
<5	1275	52.56
5∼	313	12.90
10∼	377	15.54
20∼	346	14.26
30∼	115	4.74

**Table 3:** Item scores in professional identity, job satisfaction, work engagement, and turnover intention.

Items	Mean ± SD
Professional identity	11.56 ± 2.36
My work has a great impact on the lives of others.	3.47 ± 1.13
The quality of my work will affect many people	3.89 ± 1.02
My work is very meaningful and important	4.20 ± 0.85
Job satisfaction	36.58 ± 7.12
The work itself	4.89 ± 1.09
Advancement	4.12 ± 1.50
Compensation	3.97 ± 1.44
Environment	4.49 ± 1.24
Facility	4.24 ± 1.28
Personal satisfaction	4.69 ± 1.04
Superiors	5.01 ± 1.02
Colleagues	5.16 ± 1.03
Work engagement	62.32 ± 22.09
Work vigor	21.43 ± 7.93
Work dedication	21.64 ± 8.23
Work absorption	19.26 ± 6.78
Turnover intention	10.22 ± 5.49
Thought of leaving the organization you served now	2.65 ± 1.52
Thought of leaving this industry	2.64 ± 1.53
Looking for a new job recently	2.62 ± 1.56
Looking for a new job next year	2.31 ± 1.45

**Table 4:** Correlation coefficients among study variables.

Items	Professional Identity	Job Satisfaction	Work Engagement	Turnover Intention
Professional identity				
Job satisfaction	0.35 *			
Work engagement	0.35 *	0.46 *		
Turnover intention	−0.13 *	−0.34 *	−0.26 *	

* *p* < 0.01.

**Table 5:** Significance test of the mediating test.

Model Pathways	Estimated	95% CI
Total effects		
Work engagement ← Professional identity	0.62	0.57–0.67
Job satisfaction ← Professional identity	0.68	0.62–0.73
Turnover intention ← Professional identity	−0.25	(−0.31)–(−0.20)
Turnover intention ← Work engagement	−0.13	(−0.20)–(−0.07)
Turnover intention ← Job satisfaction	−0.38	(−0.46)–(−0.30)
Direct effects		
Work engagement ← Professional identity	0.62	0.57–0.67
Job satisfaction ← Professional identity	0.68	0.62–0.73
Turnover intention ← Professional identity	0.09	(−0.02)–0.20
Turnover intention ← Work engagement	−0.13	(−0.20)–(−0.07)
Turnover intention ← Job satisfaction	−0.38	(−0.46)–(−0.30)
Indirect effects		
Turnover intention ← Professional identity	−0.34	(−0.43)–(−0.27)

**Table 6:** Significance test of every mediating pathway.

Model Pathways	95% CI
Turnover intention ← Job satisfaction ← Professional identity	(−0.64)–(−0.40)
Turnover intention ← Work engagement ← Professional identity	(−0.25)–(−0.10)
